# Should I stop or should I go on? Disease modifying therapy after the first clinical episode of multiple sclerosis

**DOI:** 10.1007/s00415-020-10074-4

**Published:** 2020-09-14

**Authors:** Tobias Monschein, Sabine Salhofer-Polanyi, Patrick Altmann, Tobias Zrzavy, Assunta Dal-Bianco, Gabriel Bsteh, Paulus Rommer, Thomas Berger, Fritz Leutmezer

**Affiliations:** Medizinische Universitat Wien, Wien, Austria

## Abstract

**Introduction:**

Treatment with disease-modifying therapies (DMT) in patients with clinically isolated syndrome (CIS) represents standard care in multiple sclerosis (MS) patients nowadays. Since a proportion of patients may show no evidence of disease activity (NEDA) after some time of treatment, the question might arise about the risks of stopping DMT.

**Methods:**

We present a cohort of 49 patients who started DMT immediately after CIS and had no evidence of disease activity (NEDA-3) for at least five years before discontinuation of therapy. Thereafter, patients underwent clinical and MRI follow-up for at least five consecutive years.

**Results:**

Of 49 patients discontinuing DMT, 53% (*n* = 26) had NEDA for at least further five years, while 47% (*n* = 23) showed either a relapse/disease progression (18.4%, *n* = 9), MRI activity (14.3%, *n* = 7) or both (14.3%, *n* = 7). The main predictive factor for sustained NEDA was age at DMT termination. Patients aged > 45 years had a significantly lower risk of disease reactivation (13% vs. 54% in patients aged < 45 years, *p* < 0.001) after DMT discontinuation.

**Discussion:**

In CIS patients with immediate DMT after their first clinical episode, older age at the time of DMT discontinuation is the main predictive factor for sustained NEDA status.

## Introduction

A clinically isolated syndrome (CIS) is defined as the first clinical symptom suggestive of inflammatory demyelination of the central nervous systems (CNS) without evidence for dissemination in time and space that are both necessary for a diagnosis of multiple sclerosis (MS) [1]. Natural history studies have shown that the majority of patients ultimately convert to clinically definite MS (CDMS) [2, 3]. Younger age at onset, male sex, type of clinical symptoms at onset [2], presence of oligoclonal bands (OCB) in cerebrospinal fluid (CSF) [4], and magnetic resonance imaging (MRI) T2-lesion load [5] are the most important prognostic factors for this conversion. CIS and MS are diagnosed using the McDonald criteria, which were recently revised in 2017, resulting in increased sensitivity and consequently lower incidence of CIS [6].

In randomized controlled trials, disease-modifying therapies (DMT) like interferon beta (IFN-beta) products and glatiramer acetate (GA) were shown to delay the conversion from CIS to CDMS significantly [7–10]. Consequently, IFN-beta and GA have been approved for CIS and are, thus, widely used in clinical routine [11].

However, prolonged periods of absence of disease activity may prompt consideration of DMT discontinuation in CIS patients and treating neurologists, especially if patients experience adverse events or syringe fatigue [12]. While the benefit–risk profiles of IFNb and GA are generally favourable, side effects, matters of convenience, and even economic burden, including costs for health care systems, may be considered in the discussion whether to continue or to stop DMT [13].

Robust data supporting the discontinuation of DMT are scarce to date, with a lack of evidence especially in clinically stable CIS patients. Natural history data suggest that inflammatory activity declines with age [14] and there is some evidence in CDMS from observational databases [12, 15, 16] or unselected retrospective cohorts [17] with the limitation of considerable heterogeneity concerning both patient population as well as reasons for DMT discontinuation (ranging from pregnancy, lack of adherence, and side effects to stable disease course).

The primary goal of our study was to evaluate possible predictive factors for NEDA status after stopping DMT in a homogenous cohort of CIS patients, who started DMT after the very first clinical episode suggestive of MS and discontinued DMT after remaining free of disease activity for at least five years.

### Patients and methods

**Fig. 1:**
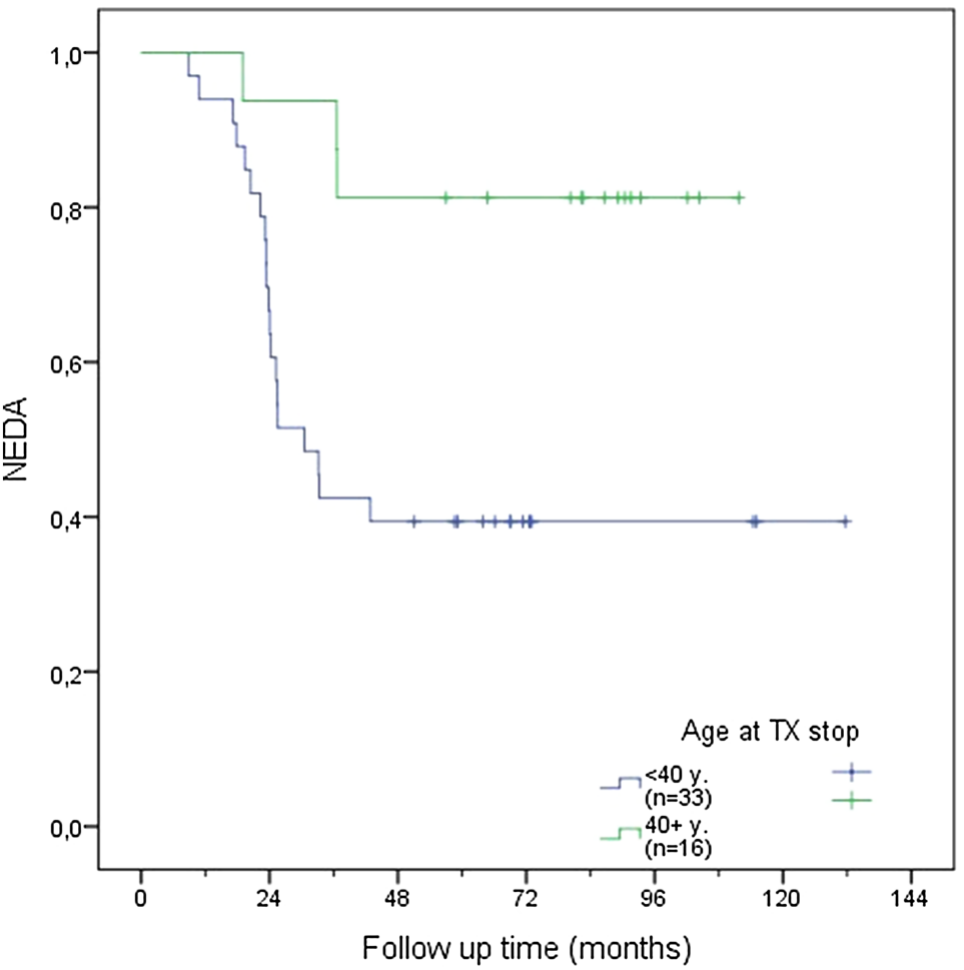
NEDA rate according to age above or below age of 40 years at DMT discontinuation

This is a retrospective analysis of prospectively collected observational data. We recruited patients from the MS outpatient clinic of the Department of Neurology, Medical University of Vienna, diagnosed between 2001 and 2011 with a CIS according to McDonald criteria 2001 (see Fig. 1). The study was approved by the ethics committee of the Medical University of Vienna (EK 1203/2016).

Inclusion criteria were initiation of DMT (IFN-beta or GA) after a diagnosis of CIS was established. Even though the 2017 revision of the McDonald diagnostic criteria are the generally accepted criteria for MS diagnosis nowadays, for the purpose of this work, it is important to emphasize that we used the 2001 McDonald diagnostic criteria (applicable when the majority of the patients from our cohort was diagnosed). This version defined CIS as a monosymptomatic event suggestive of a first inflammatory demyelinating event with an acute onset reaching a peak within 14 days in the absence of objective clinical evidence of a second lesion and without evidence for dissemination in time and space as derived from typical MRI findings and eventually from positive oligoclonal bands in the cerebrospinal fluid (CSF) [1]. The risk of misdiagnosis at this very early stage of the disease was minimized using the more conservative Barkhof MRI criteria in all patients, and CSF testing for oligoclonal bands (OCB). The Barkhof criteria consist of at least 1 gadolinium-enhancing lesion or at least 9 lesions on T2-weighted images, at least 3 periventricular lesions, at least 1 juxtacortical lesion and at least 1 infratentorial lesion. 3 of the 4 variables must be met [18].

After initiation of DMT, patients had to have no evidence of disease activity (NEDA-3) for at least five consecutive years before discontinuation of therapy [19]. In this case, the option of DMT discontinuation was discussed with the patient extensively; however, the decision was primarily based on the patient’s individual choice. After discontinuation of DMT, patients underwent regular clinical examinations at least annually (clinical history with documentation of confirmed relapse and disease progression as measured by the Expanded Disability Status Scale (EDSS)) as well as annual MRI follow-ups for at least five consecutive years.

A relapse was defined as a typical symptom of an acute inflammatory demyelinating event lasting for at least 24 h, at least 30 days apart from the last episode, and without associated temperature increase or recent infection.

Furthermore, a confirmed sustained EDSS increase of 0.5 or more at 6-month follow-up as compared to baseline (i.e. time of treatment initiation) was defined as EDSS progression. MRI activity was defined as either a new or enlarging T2 lesion or a new contrast-enhancing lesion as compared to a prior MRI. NEDA-3 was defined as the absence of relapse, disease progression and MRI activity [19].

Patients were divided into two groups: the first group was labelled “evidence of disease activity” (EDA) and included those patients who suffered either a relapse, EDSS progression, and/or MRI activity during the 5-year follow-up period; the second group was called "NEDA" comprising patients with no evidence of disease activity (NEDA-3) within five years of follow-up. In addition, various age cut-offs were analysed based on the median of the group (31y) and current available data with regard to the NEDA rate (40y and 45y) [12].

### Statistics

The statistical evaluation of the collected data was done in SPSS (SPSS Inc. Version 26.0, Chicago, IL, USA). Continuous parametric variables were tested for normal distribution by Kolmogorov–Smirnov test. Categorical variables were expressed in frequencies and percentages, continuous parametric variables as either mean and 95% confidence intervals (95% CIs) or median and range as appropriate depending on normal distribution. The primary endpoint of the study was the comparison of EDA versus NEDA after a minimum follow-up of at least five years.

Univariate differences between patient groups in categorical variables were evaluated using cross-tabulation and chi-square test corrected by Fisher's exact test. Numeric variables were analysed by independent *t*-test or Mann–Whitney *U* test as appropriate depending on normal distribution.

Multivariate analyses were performed using binary logistic regression. Group comparisons regarding time to event (NEDA-3 status) were investigated using Kaplan–Meier curves and Cox regression models. The log-rank test was used for evaluation of significance. The significance level was set with a *p* < 0.05 (two-sided).

## Results

We included a total of 49 patients. The mean age at initial symptoms was 26.3 ± 7.5 years and 65% were female. Demographic and clinical characteristics are given in Tables 1 and 2. Table 1 additionally shows our data in comparison to the baseline characteristics of the large phase III clinical CIS trials.

**Table 1:** Comparison of demographical, clinical and paraclinical data in CIS studies (BENEFIT; CHAMPS; PRECISE; REFLEX) compared to this study

	BENEFIT [20]	CHAMPS [21]	PRECISE [9]	REFLEX [10]	Own Cohort
Treatment	SC IFNß-1b	IM IFNß-1a	GA	SC IFNß-1a	various
Age (years)	30	33	31	31	26
Female (%)	71	73	65	67	65
HDMP for first demyelinating event (%)	71	nk	67	71	96
Monofocal symptoms (%)	52	nk	nk	56	92
NNO (%)	29	49	nk	nk	33
Brainstem/Cerebellar (%)	22	30	nk	nk	18
Spinal (%)	34	21	nk	nk	5
Cerebral (%)	15	nk	nk	nk	44
Multifocal symptoms (%)	48	nk	nk	nk	8
EDSS	1.5	nk	1.0	nk	1.0
Patients with CSF analysis (%)	68	nk	nk	nk	100
OCB positivity (%)	59	nk	nk	nk	88
Number of T2 lesions ≥ 9 (%)	71	28 (≥ 8)	84	72	78
CEL ≥ 1 (%)	43	20	47	40	43
Time from onset to DMT start (days)	nk	20	79	nk	83

*GA* Glatirameracetate. *IM IFNß-1a* intramuscular Interferon beta-1a, *SC IFNß-1b* subcutaneous Interferon beta-1b, *SC IFNß-1a* subcutaneous Interferon beta-1a, *HDMP* high-dose methylprednisolone, *NNO* neuritis nervi optici, *EDSS* expanded disability status scale, *CSF* cerebrospinal fluid, *OCB* oligoclonal bands, *CEL* contrast-enhancing lesion, *DMT* disease-modifying therapy, *nk* not known

Demographic data among the various studies show comparable distributions. Although the proportion of monofocal and multifocal onset symptoms is distributed differently, the diagnostic certainty in our cohort is, however, supported by cerebrospinal fluid analyses in all our patients, demonstrating positive oligoclonal bands in 88% of patients.

**Table 2:** Cohort characteristics

	Total (*N* = 49)	EDA (*n* = 23)	NEDA (*n* = 26)	*p*-value (EDA vs NEDA)
Age at (years)^1^				
Initial symptoms	26.3 ± 7.4	22.7 ± 5.2	29.5 ± 7.7	0.001^4^
DMT start	26.5 ± 7.5	22.9 ± 5.3	29.8 ± 7.8	0.001^4^
DMT stop	33.9 ± 9.0	30.9 ± 7.0	37.4 ± 9.3	0.003^4^
Female^2^	32 (65.3)	14 (60.9)	18 (69.2)	0.564^6^
DMT^1^				
Time to start (months)	2.7 ± 1.9	2.4 ± 1.8	3.0 ± 1.9	0.214^4^
Time on DMT (years)	7.4 (2.5)	7.1 ± 2.3	7.6 ± 2.6	0.484^4^
EDSS^3^				
At DMT start	1 (0 – 3)	1 (0 – 2)	1 (0 – 3)	0.730^5^
At DMT stop	0 (0 – 2)	0 (0 – 2)	0 (0 – 1)	0.088^5^
OCB positivity^2^	43 (87.8)	21 (91.3)	22 (84.6)	0.476^6^
Initial MRI^2^				
CEL present	21 (42.9)	10 (43.5)	11 (42.3)	0.934^6^
Spinal cord lesions	21 (42.9)	10 (43.5)	11 (42.3)	0.934^6^

^1^Mean and standard deviation

^2^Number and percentage

^3^Median and range. *p*-values calculated for comparison of EDA and NEDA group using

^4^Independent-test

^5^Mann-Whitney-*U*-test or

^6^Chi-square-test

*CEL* contrast-enhancing lesion, *DMT* disease-modifying therapy, *EDA* evidence of disease activity, *MRI* magnetic resonance imaging, *NEDA* no evidence of disease activity, *OCB* oligoclonal bands

26 (53%) of 49 patients presented with sustained NEDA at 5-year follow-up after having had discontinued DMT. In contrast, 47% (*n* = 23) showed signs of clinical and/or MRI disease activity. Clinical (relapse and or disease progression) or MRI activity, or a combination of both was found in 18.4% (*n* = 9), 14.3% (*n* = 7), and 14.3% (*n* = 7), respectively.

Age at DMT discontinuation was significantly higher in the NEDA group (*p* = 0.003), which was the only factor with significant difference. However, there was also a tendency (*p* = 0.088) to a lower EDSS in the NEDA group (see Table 2). On the other hand, sex, type of DMT, presence or absence of OCB, CELs or spinal cord involvement on initial MRI, treatment duration, and CIS symptom (*p* = 0.774) showed no association with NEDA status.

In the multivariate analysis, by binary logistic regression, only age at discontinuation had a significant influence with higher age associated with higher probability of NEDA status. In the Cox regression as a function of time (until EDA or last follow-up), older age was significantly associated with lower risk of EDA, while female sex only showed a very modest association (Tables 3 and 4).

The mean time to risk of EDA was significantly shorter in patients younger than 31 years (26 months, *p* = 0.006) compared to 40 years (31 months, *p* = 0.004) and 45 years (37 months, *p* = 0.044).

**Table 3:** Multivariate analysis by use of binary logistic regression regarding EDA versus NEDA; for therapy analysis GA was chosen as reference and compared to the different interferon products

Predictor	*B*	SE	Wald χ^2^	df	*p*	OR	95% CI OR	
							LL	UL
Sex (female)	1.258	0.851	2.184	1	0.139	3.518	0.664	18.650
Age (stop)	– 0.176	0.056	9.770	1	0.002	0.838	0.751	0.936
OCB (positivity/ negativity)	1.435	1.138	1.590	1	0.207	4.198	0.452	39.024
CEL (Yes/No)	– 0.880	0.843	1.089	1	0.297	0.415	0.079	2.166
Spinal MRI lesions (Yes/No)	0.185	0.752	0.061	1	0.805	1.204	.276	5.258
Therapy (GA)			0.691	3	0.875			
IM IFNß-1a (30mcg)	0.522	0.918	0.324	1	0.570	1.686	0.279	10.197
SC IFNß-1b (250mcg)	– 0.144	1.107	0.017	1	0.897	0.866	0.099	7.576
SC IFNß-1a (44mcg)	– 0.324	0.985	0.108	1	0.742	0.723	0.105	4.989

*CEL* contrast-enhancing lesion, *GA* Glatirameracetate, *IM IFNß-1a* intramuscular Interferon beta-1a, *SC IFNß-1b* subcutaneous Interferon beta-1b, *SC IFNß-1a* subcutaneous Interferon beta-1a

**Table 4:** Cox regression in dependence of time (until relapse or last follow-up) regarding EDA versus NEDA; for therapy analysis GA was chosen as reference and compared to the different interferon products

Predictor	*B*	*SE*	Wald χ^2^	df	*p*	HR	95% CI HR	
							LL	UL
Sex (female)	1.083	.545	3.954	1	0.047	2.955	1.016	8.597
Age (stop)	– 0.164	.042	15.010	1	< 0 .001	0.849	0.781	00.922
OCB (positivity/ negativity)	0.676	0.824	0.673	1	0.412	1.967	0.391	9.894
CEL (Yes/No)	– 0.404	0.497	0.661	1	0.416	0.668	0.252	1.769
Spinal MRI lesions (Yes/No)	– 0.077	0.505	0.023	1	0.0878	0.926	0.344	2.491
Therapy (GA)			1.482	3	0.686			
IM IFNß-1a (30mcg)	0.443	0.578	0.586	1	0.444	1.557	0.501	4.832
SC IFNß-1b (250mcg)	0.375	0.705	0.282	1	0.595	1.454	0.365	5.790
SC IFNß-1a (44mcg)	– 0.240	0.611	.154	1	0.694	0.787	0.238	2.605

*CEL* contrast-enhancing lesion, *GA* Glatirameracetate, *IM IFNß-1a* intramuscular Interferon beta-1a, *SC IFNß-1b* subcutaneous Interferon beta-1b, *SC IFNß-1a* subcutaneous Interferon beta-1a

**Fig. 2:**
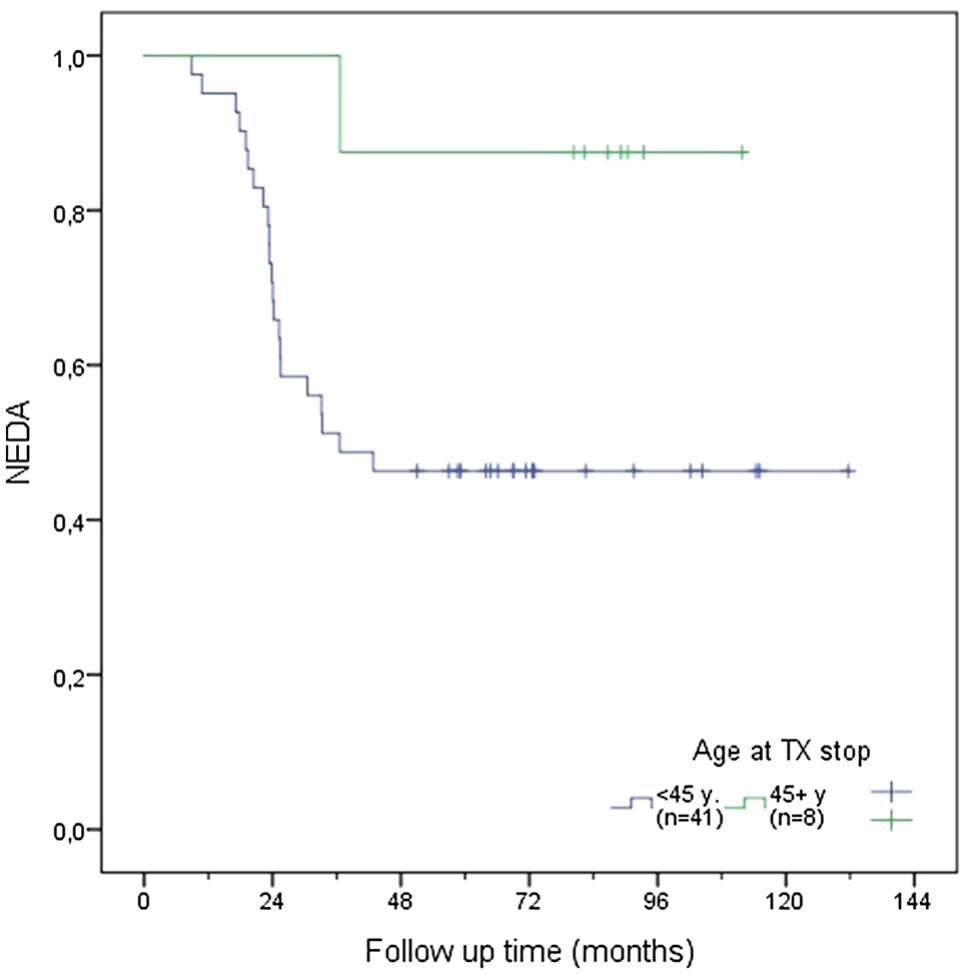
NEDA rate according to age above or below age of 45 years at DMT discontinuation

**Fig. 3:**
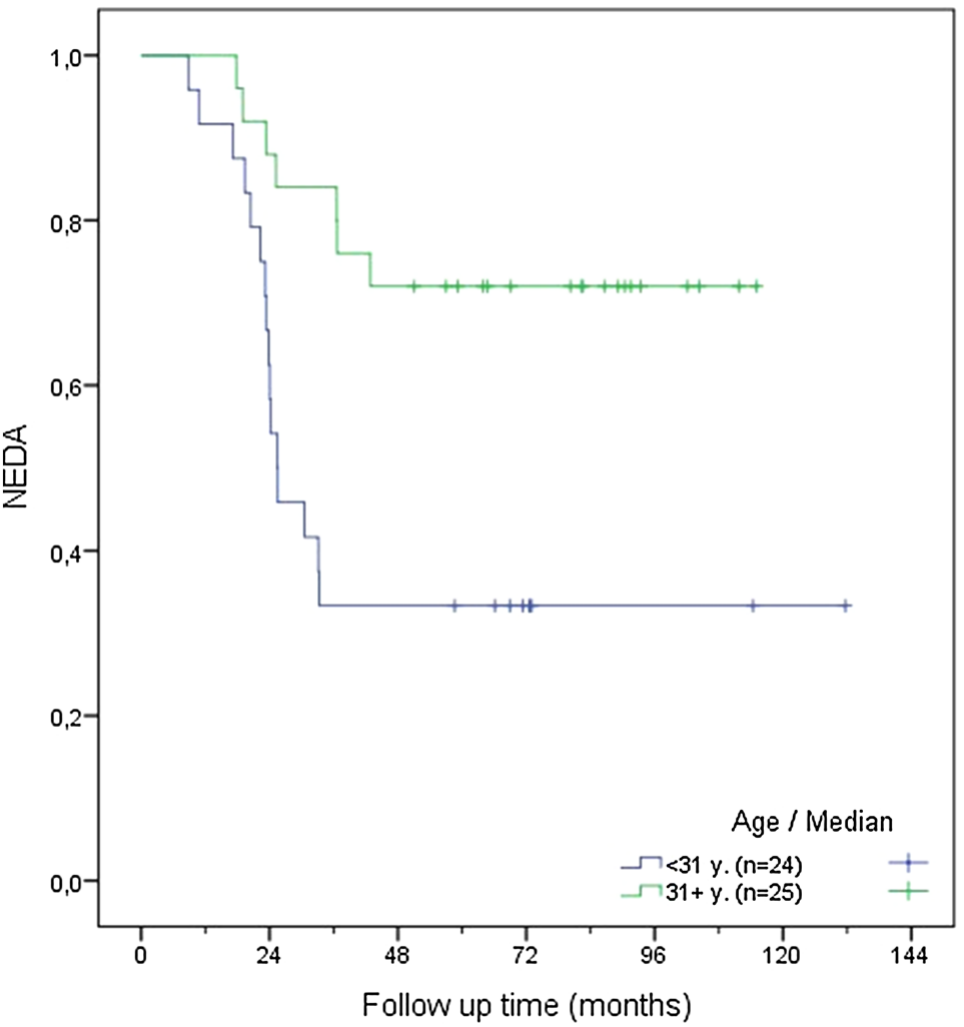
NEDA rate according to age above or below the median age of the total group (31 years) at DMT discontinuation

In a further step, time to EDA was compared according to different age cut-offs (31, 40 and 45 years) using the Kaplan–Meier function (see Figs. 1, 2, 3). Patients older than 40 years at DMT discontinuation had a significantly lower risk of recurrence of disease activity compared to those younger than 40 years (see Fig. 1; 18.8% vs. 60.6%, *p* = 0.006). A similar difference was found when comparing patients aged > 45y/ < 45y (see Fig. 2; 12.5% vs. 53.7%, p = 0.025) and > 31y/ < 31y (see Fig. 3, *p* = 0.003, 28% vs. 66.7%).

## Discussion

To our knowledge, this is the first study determining the risk of disease recurrence after DMT discontinuation in a cohort of patients, comparable to study populations of former phase III clinical trials in CIS, who started DMT immediately after CIS and remained NEDA-3 for at least 5 years thereafter.

In this cohort, older age at the time of DMT discontinuation was the main predictive factor for sustained NEDA after discontinuation. Patients aged > 45 years had a significantly lower risk for disease reactivation (13% vs. 54%, *p* < 0.001) after DMT discontinuation. Furthermore, female gender was also shown to be a significant positive predictive factor for NEDA, although the small sample size (*n* = 49), level of significance (*p* = 0.047) and the lower limit of Hazard-Ratio (HR) (1.016) must be considered. All other variables (type of DMT, presence or absence of OCB, CELs, spinal cord involvement on initial MRI, treatment duration, and CIS symptom) did not show predictive relevance for disease reactivation after DMT discontinuation.

Our results are in line with large, multi-centre registry-based cohorts [15, 16] as well as single-centre cohorts [12, 17] of more heterogeneous patient groups, both with respect to the time of treatment initiation as well as the reason of DMT discontinuation.

In contrast to the above-mentioned studies, our cohort comprised only patients starting their DMT after clinical disease onset. Importantly, our cohort had a very low risk of MS misdiagnosis due to application of Barkhof’s strict MRI criteria in all participants as well as the presence of OCBs in CSF in the vast majority of patients (88%). Moreover, the presence of positive OCB, CEL and spinal MRI lesions at the time of diagnosis was similarly distributed between NEDA and EDA patients, arguing against the possibility that NEDA patients may have had a higher risk of MS misdiagnosis.

In CDMS, Kister et al. found similar relapse rates in patients who stopped their DMT after a relapse-free period of at least 5 years as compared to those patients who remained on therapy [15]. However, the risk of post-DMT relapse was higher in younger patients, consistent with natural history studies, showing a decline in relapse rate with age [22]. In contrast, the risk of disability progression in this cohort increased with age, again in accordance with natural history data [23].

Nevertheless, the risk of disability progression was higher in DMT stoppers as compared to patients with ongoing DMT use. This could be at least in part due to the fact that DMT discontinuation was more likely in older patients with a higher “natural” risk of conversion to SPMS.

From the same but extended cohort, Kister et al. [16] proposed a higher risk of post-DMT relapse in younger patients and females. This sex difference was vice versa in our cohort as already mentioned above. However, the effect sizes are very low rendering clinical relevance very unlikely. Similar results were also gained in a single-centre retrospective analysis of 221 prospectively followed RRMS patients who stopped DMT due to a variety of reasons [12]. Age ≥ 45 years at discontinuation, absence of relapses for ≥ 4 years on DMT before discontinuation, and absence of CELs on MRI were independent predictors of ongoing freedom of disease activity. Based on these data, we chose the cut-offs of 40 and 45 years in our analyses and cut off 45 showed the highest NEDA rate.

The strength of our study is the data completeness in terms of clinical and MRI data at baseline as well as during and post-DMT and the fact that DMT withdrawal was exclusively due to NEDA. It is important to point out that this is an essential difference to other studies where reasons for discontinuation were quite heterogenous [12, 15, 16]. In contrast to these studies, who included patients with DMT initiation ranging from CIS to late-stage RRMS and even SPMS, we aimed to specifically address the question of DMT discontinuation in the increasing population of patients with DMT initiation after the very first clinical episode.

Especially in this patient group, both, patients and treating neurologists, might face the question “should we stop or go on with DMT?” Factors that heat up this discussion might be inconvenience, side effects, costs, but also the possibility of a favourable disease course, which was already reported in the pre-DMT era at least in a small proportion of patients [24]. Thus, not all patients might be in need of a long-term DMT without hampering long-term prognosis [25].

Our study has several limitations. First, sample size was small, and we do not have a control group. Furthermore, our patients were classified as CIS according to the McDonald criteria 2001. Considering that 43 of 49 patients had positive OCBs, and of those with negative OCBs, 5/6 had CELs on initial MRI, 48/49 patients would have been classified as definite MS according to the current McDonald criteria [1, 6].

In conclusion, in a homogenous group of CIS patients who started DMT immediately after the first clinical event and discontinued after achieving at least 5 years of NEDA, we could identify age at DMT discontinuation as the main predictive factor for re-occurrence of disease activity. These data should not encourage CIS patients to generally stop DMT after a long NEDA period. However, it could provide some support to physicians in advising patients, who do no longer want to continue DMT because of sustained freedom of disease activity. In this context, neurologists should encourage younger people to continue DMT despite no overt clinical or MRI indicators of ongoing disease activity, while in patients > 45 years, stopping DMT together with continuous regular clinical and MRI follow-up may provide a clinically reasonable option with low risk.
